# Youth Are the Experts! Youth Participatory Action Research to Address the Adolescent Mental Health Crisis

**DOI:** 10.3390/healthcare12050592

**Published:** 2024-03-05

**Authors:** Kimberly E. Smith, Rosa Acevedo-Duran, Jennifer L. Lovell, Aliyah V. Castillo, Valeria Cardenas Pacheco

**Affiliations:** 1 Department of Health, Human Services, and Public Policy, California State University Monterey Bay, Seaside, CA 93955, USA; kimsmith@csumb.edu; 2 Department of Psychology, California State University Monterey Bay, Seaside, CA 93966, USA; racevedoduran@csumb.edu; 3 Gonzales Youth Council, Gonzales, CA 93926, USA

**Keywords:** youth participatory action research, COVID-19 pandemic, adolescent mental health, community-based participatory research

## Abstract

Adolescent mental health is an urgent global public health issue. Youth participatory action research is an effective strategy to amplify youth voices and can serve as a catalyst for evidence-based action addressing the mental health crisis. To illustrate the benefits of youth participatory action research for informing community health, we describe an ongoing collaboration with a youth council located in the central coast of California, USA. Research methods included an anonymous online self-report survey to gather information about the mental health of high school students in 2020 (*n* = 176) and 2022 (*n* = 234), 93% Latinx/Mexican American. Both surveys included a four-item patient health questionnaire to screen for depression and anxiety risk, in addition to scaled and open-ended survey questions selected by the youth leaders based on their research questions. Quantitative and qualitative results indicated a significant but small decrease in mental health risk, and a continued need for resources to access mental health support. Results led to community-based action aimed at improving local youth mental health. The interdisciplinary research team (psychology and public health) and youth leaders share reflections highlighting the innovative, empowering, and transformative impact of youth participatory action research as a tool for improving community health.

## 1. Introduction

The devastating rise in adolescent mental health challenges is an urgent public health issue in need of timely research and action [1]. The COVID-19 pandemic created multiple threats to youth mental health with more than a third of youth experiencing poor mental health during the pandemic [2], and ethnic/racial minority youth and youth living in poverty were at a higher risk for poor mental health outcomes [1,3]. With many influences shaping the lives of adolescents, a holistic and system-based approach to solutions is necessary for positive health and wellness. Improved support for youth mental health is possible by intervening in the many institutions surrounding young people [1]. One research tool to help address the youth mental health crisis is youth participatory action research (YPAR), a collaborative research approach utilizing youth as primary investigators [4]. Youth voices are leveraged in the research process to spur positive social change and empowerment by identifying and investigating important issues within their communities. Participatory action research is considered both a collaborative methodology and a theoretical agenda [5], with transformative and critical-ideological theoretical paradigms underpinning the research process [6]. Based on the literature and our prior YPAR work [7], some framing principles of YPAR are outlined in Table 1.

The YPAR research approach places young people as primary investigators in the research process to study and improve conditions in different settings relevant to their lives, such as schools, neighborhoods, or health systems [8]. Consistent with the principles outlined in Table 1, it is optimal when the YPAR process can be iterative and embedded within larger systems of care [9], such as the healthcare system. YPAR involves a cycle of research, action, and evaluation in which youth advocate for changes informed by their research findings. It is a research-intensive form of youth participation in advocacy and community change, with a strong emphasis on young people’s active involvement in decision-making related to the research process.

In this study, we used YPAR to investigate youth mental health through an ongoing partnership with a youth council in a city with a predominantly Latinx population. We contextualize this work by first reviewing the literature on Latinx and adolescent mental health and YPAR within the healthcare field. We then describe details about our YPAR project in which the research design, recruitment, analysis, and interpretation were youth-led with support from University partners. Lastly, we discuss the power of YPAR research in addressing community health issues, Latinx youth mental health, and implications for future healthcare studies.

### 1.1. Latinx and Adolescent Mental Health

The Latinx population is the largest and fastest-growing ethnic minority population in the United States (U.S. or USA), making up 19.1% of the total population [10]. In this paper, we use Latinx as a gender-neutral term to describe individuals of Latin origin [11], but we also use Hispanic when the term is used within other publications. People who identify as Hispanic or Latinx are individuals of Cuban, Mexican, Puerto Rican, South or Central American, or other Spanish culture or origin, regardless of racial identity [12]. This term is imperfect, and there are limitations to using this term to categorize a large and diverse group of people. Youth leaders originally part of the present YPAR project identified as Latinx with Mexican origin, and currently, many of the youth leaders identify as Mexican American.

Though one of the fastest-growing minority populations, the Latinx community continues to face significant barriers to utilizing mental health support and increased risk for some mental health disorders [12,13]. Some of the barriers include 16.8% of the U.S. Latinx population having no insurance coverage and 14.3% of families and persons living beneath the poverty line [14]. Additional barriers to accessing mental health care include mental health stigmas, fears related to legal status, and lack of awareness of accessible health and mental health care services [13]. Regarding citizenship, 66.3% of Hispanics are native U.S. citizens, 33.7% are foreign-born, with 13.0% being naturalized citizens and 20.6% being non-U.S. citizens [15]. By 2060, the U.S. Census Bureau estimates the Hispanic population will grow to 26.9% of the U.S. population [16]. In 2022, educational attainment rates indicated that 26.9% of Latinx respondents had less than a high school diploma, and 28.1% were high school graduates [17]. The young population of Hispanics and Latinx has shown a dramatic increase in educational attainment in recent decades, with an increase in high school graduates and college enrollments [17].

Due to the complex identities and challenges facing the Latinx population in the U.S., it is essential to view mental health challenges within the cultural context. Adolescence is a formative development period marked by social and biological changes. Risks for anxiety and depression are prominent among adolescents due to the physical, social, and emotional changes during this phase of development, which can be heightened by exposure to traumatic experiences, poverty, and discrimination [18]. Hispanic/Latinx adolescents confront additional stressors besides the normative stress of adolescence that may heighten mental health risk and pose cultural, linguistic, and socioeconomic barriers [19]. For example, experiencing acculturative stress and discrimination can increase the risk of developing depression and anxiety [19]. Latinx youth may also be less likely to receive mental health services due to cultural beliefs and responsibilities [20]. Culturally relevant mental health treatment is needed among many Latinx youth and families due to limited access, knowledge, and bilingual or linguistically trained mental health professionals [13].

Depressive and anxiety symptoms doubled among youth during the first year of the COVID-19 pandemic based on a meta-analysis including over 80,000 young people globally (approximately 25% reporting depressive symptoms and 20% reporting anxiety symptoms) [21]. According to the 2023 Youth Risk Behavior Survey data summary from 2011–2021, Hispanic and multiracial students were more likely to have persistent feelings of sadness or hopelessness over time [22]. In combination with increased mental health risks during the pandemic, high schoolers also reported a decreased sense of well-being, with disproportionate impacts on Latinx, women, nonbinary youth, and youth experiencing food insecurity [23]. Depressive disorders are marked by the presence of a sad, empty, or irritable mood [24]. Depression is a prevalent mood disorder among adolescents, with higher rates among low and middle-income communities [25]. Anxiety disorders are marked by the features of excessive fear, anxiety, and disturbances to behavior, differing from normative fear and anxiety due to the excessiveness and persistence beyond an appropriate period [24]. Anxiety disorders are also common disorders affecting children and adolescents [26]. In contrast to mental disorders and symptoms, well-being is characterized by positive health and flourishing; Seligman [27] identified five pillars of well-being, including positive emotions, engagement, relationships, meaning, and accomplishment. Addressing Latinx youth mental health and wellbeing is essential for youth and their communities to thrive.

### 1.2. Youth Participatory Action Research in Healthcare

Few studies on youth mental health are initiated by, led, and co-authored by youth. However, there is an increasing call for youth participation and engagement around issues that impact youth, and YPAR can be a powerful tool for healthcare professionals across disciplines. YPAR is a research approach that actively involves young people in research processes, empowers them to drive change in their communities and systems, and has the potential to enhance the effectiveness and relevance of interventions and research aimed at improving the well-being of young people across the lifespan [8]. This research approach places young people as primary investigators in the research process to study and improve conditions in different settings relevant to their lives, such as schools, neighborhoods, or health systems [8]. Consistent with the principles outlined in Table 1, it is optimal when the YPAR process can be iterative and embedded within larger systems of care [9]. YPAR involves a cycle of research, action, and evaluation in which youth advocate for changes informed by their research findings. It is a research-intensive form of youth participation in advocacy and community change, with a strong emphasis on young people’s active involvement in decision-making related to the research process. Civic engagement can also serve as a means of coping with social injustices for youth from marginalized communities. Youth civic action has been found to promote critical thinking skills, community engagement, and social and emotional learning skills [18].

YPAR is aligned with the principles of life course intervention research as it focuses on empowering young people to respond to the stressors they experience and improve the systems that affect their healthy development [8]. YPAR can help shape life course development by addressing social, racial, and economic inequities that affect young people’s health and well-being. It also offers an equity-focused, anti-racist lens by challenging dominant narratives and involving young people as experts in addressing structural racism and promoting health equity in their communities.

Having youth engage in research on youth mental health and wellbeing is also a way to improve and support adolescent *mental health literacy*, an important concept in public health, psychiatry, and prevention. There are various definitions, but Kutcher et al. [28] defined mental health literacy with four domains, including: “understanding how to obtain and maintain positive mental health; understanding mental health disorders and their treatments; decreasing stigma related to mental disorders, and enhancing help-seeking efficacy” (p. 155). Spiker and Hammer [29] argued for reconceptualizing mental health literacy as a “multi-construct theory, rather than a multi-dimensional construct” (p. 3), allowing for a better delineation of the domains and how they can be used to support adolescent mental health [30]. These domains can help inform research on youth mental health and the impact of YPAR on health literacy among youth.

YPAR is an approach that can be used by health educators and public health professionals to promote skill development among diverse young people [4,31] while improving systems of care and quality of life across healthcare settings. For example, YPAR projects can improve the validity and meaningfulness of research questions and outcomes [32]. Thus, YPAR shows promise as a tool for involving youth in public health planning and fostering community change driven by youth [31]. However, further research is required to establish causal connections between YPAR program principles, processes, and outcomes in healthcare settings. Youth engagement has been recognized as a “best practice” in public health education and is promoted by the Centers for Disease Control and Prevention [33], but is not often seen in the public health literature. A previous YPAR project [7] in our target community demonstrated how the YPAR process of research, participation, and transformation can be used as a strategy to advocate for local youth mental health services.

### 1.3. The Current Project

This study is a 2022 follow-up to a 2020 YPAR project focused on youth mental health amidst the COVID-19 pandemic (see Rocha et al. [7] for details of partnership and 2020 project). The present project is an ongoing collaboration between University partners and the Gonzales Youth Council (GYC)—a youth-led organization in a predominantly Latinx/Mexican-American agricultural community in Monterey County, CA. The University partners include an interdisciplinary team, with psychology and public health undergraduate students under the mentorship of a psychology professor (J.L.L.). The GYC was established in 2015 through a partnership between the City of Gonzales and the Unified School District to increase youth voice and action within their community. The GYC includes approximately 15 youth (middle and high school students) led by 2–3 youth commissioners and 2–3 ambassadors. The group leaders and members shift yearly, but some youth continue to serve for multiple years. In 2020, the GYC youth leaders identified research question(s) and connected with a University professor (J.L.L) to conduct community-based participatory research.

The 2020 GYC used their research to investigate students’ mental health in May 2020 (during remote education due to the COVID-19 pandemic), and they used their findings to engage in local activism and advocacy for youth mental health. The 2021–2022 GYC continued this collaboration and conducted a follow-up survey in May 2022. This phase of the project aimed to continue identifying and advocating for the mental health needs of youth in Gonzales. The 2020 and 2022 online surveys were anonymous and included two different groups of students. We are sharing our key findings and process from the current project to promote the use of YPAR in healthcare and to demonstrate the power of research produced by youth for youth mental health.

#### Research Questions and Hypotheses

Our 2022 research questions included: (1) How does the mental health of Gonzales High School students in 2022 compare to 2020? and (2) How can we improve access to and utilization of mental health resources?

These research questions are connected to mental health literacy domains of knowledge about well-being and mental illness, knowledge of where and how to seek mental health information, and perceived barriers to help-seeking [30]. The youth hypothesized, based on their lived experiences, that wellbeing/happiness would improve and mental health risk for depression and anxiety would decrease from 2020 to 2022. However, they also still felt there were numerous unmet mental health needs for themselves and their peers. There was no formal hypothesis for the second research question since this was exploratory and qualitative.

## 2. Materials and Methods

### 2.1. Researcher and Researcher-Participant Relationships

YPAR is a collaborative research approach [4]. Our partnership began when a few GYC leaders met the university professor at an in-person networking event hosted in February 2020. We formed a meaningful connection, and the GYC contacted the university professor to collaborate on their mental health project. As the collaborative research process progressed, we developed and signed a memorandum of understanding to establish youth ownership of the data and social responsibility regarding the research process. Every presentation and publication has involved youth approval and/or involvement. Our formal collaboration began in March/April 2020 amidst the COVID-19 pandemic, lockdowns, and remote education. Our meetings and research workshops were all via video conferencing (i.e., Zoom). Starting in 2022, we began meeting in person for some workshops, events, and presentations. The GYC leaders and undergraduate researchers have shifted each year due to graduations. The university undergraduate students have usually been psychology majors, but in 2022 a student from collaborative health and human services (public health) also joined our team. Having an interdisciplinary team was particularly helpful for deepening our reflection and conceptualization of our work in the healthcare field. Two undergraduate students are the first authors of this manuscript, and two GYC youth are co-authors of this manuscript (AVC and VCP). The GYC members were high school students who were also researchers on this project, and the research participants were their peers. Please see the manuscript’s Supplemental Materials for our biographical and reflective statements that contextualize our research approach. Our identities helped inform our commitment, personal connection, interpretation, and actions based on this project.

### 2.2. Participants

The 2022 study included 234 participants from the local public high school. Students identified as male (*n* = 111, 47.4%), female (*n* = 104, 44.4%), and other/preferred not to answer (*n* = 19, 8.1%). Regarding ethnic/racial identity, the majority identified as Latino/a/x, Mexican-American, or Hispanic (*n* = 218, 93.2%). Most participants identified as 11th graders *(n* = 106, 45.3%) or 9th graders (*n* = 62, 26.5%). The 2020 sample included 176 high school students and 111 middle school students. We focus on high school students in the current study since the 2022 survey only included high school students. Of the high school students in 2020, 63.6% identified as female (*n* = 112), and 34.1% were male (*n* = 60). The majority also identified as Latino/a/x, Mexican-American, or Hispanic (92.0%; *n* = 162). School status included a high proportion of 10th graders (*n* = 67, 23.2%).

There were some significant differences between the two samples. Chi-Square Test for Independence indicated a significant association between sample cohort (2020 vs. 2022) and school grade, *χ^2^*(3, *n* = 403) = 52.8, *p* < 0.001, *Cramer’s V* = 0.36. Using the Yates’ Continuity Correction, there was also a significant association between cohort (2020 vs. 2022) and gender, *χ^2^*(1, *n* = 387) = 10.2, *p* < 0.001, *phi* = −0.17. The 2022 sample included more males and a higher proportion of 9th and 11th graders. The 2020 sample included more females and a higher proportion of 10th graders. See additional details in Table 2. 

### 2.3. Measures

The 2022 survey included 15 questions, including demographic questions. The youth selected two measures of mental health and wellness. First, the Patient Health Questionnaire (PHQ-4 [34]) is a four-item screener for depression and anxiety symptoms. This measure asks, “Over the last 2 weeks, how often have you been bothered by the following problems…” and includes a list of two depression (e.g., “Feeling down, depressed, or hopeless”) and two anxiety (e.g., “Feeling nervous, anxious or on edge”) subscale prompts. Each item is rated from 0 (*not at all*) to 3 (*several times a day*). Categories of risk are determined by cutoff scores and include: normal/mild (0–2 on either subscale, or a total score of 0–5) and moderate to severe (score of 3 to 6 on either subscale, or a total score of 6–12). The 2020 GYC selected this measure because they wanted to understand the mental health of youth, and after discussion with University partners, they felt that a validated and reliable screener would help with their advocacy efforts. The University partners provided the group with a few options, and the 2020 GYC selected the PHQ-4 because they felt it was easy to understand and short. The 2022 GYC decided to keep the PHQ-4 as part of their survey to compare their results to 2020. The PHQ-4 has been used in other high school student samples with good reliability [35,36]. Cronbach’s alpha for the PHQ-4 in the current study indicated high internal consistency within the 2020 (α = 0.85) and 2022 (α = 0.86) samples, which is similar to the original sample of the measure (Kroenke et al., 2009) [34].

Second, happiness was used as an indicator of well-being and measured with one question selected from the PERMA-Profiler [37]. The PERMA-Profiler is a general measure of flourishing, including 23 items across five domains: positive emotion, engagement, relationships, meaning, and accomplishment. One item measured overall happiness, which is the item GYC selected to include in their study. The item asked: “How happy would you say you are”? on a scale of 0 (*not at all*) to 10 (*completely*). Abdel-Khalek [38] found support for using a single-item happiness item (rated on an 11-point scale, as in this study) as a reliable and valid measure of happiness for secondary students and university undergraduates in Kuwait. In particular, the single-item demonstrated good concurrent validity with measures of happiness and life satisfaction, convergent validity with optimism and positive affect, and divergent validity with measures of anxiety and negative affect. Lukoševičiūtė et al. [39] also found adequate support for the use of a single-item happiness measure among adolescents across three European regions. The 2020 GYC originally wrote their own happiness item, and the University partners brought in this single-item question as a possible alternative. Although using only one item from a measure is not optimal, the GYC felt this was a good fit because of the scaling and simplicity of the question. The 2022 GYC decided to keep this happiness question as part of their survey because they wanted to use it to compare their results to the 2020 results.

We developed seven questions in collaboration with the 2022 GYC to measure barriers and access to mental health resources. Four of these questions were only locally relevant. One quantitative question asked, “How likely would you be to seek support from a mental health counselor if you started experiencing a mental health challenge (ex., depression, anxiety)”? This item was scaled from 1 (*not at all likely*) to 5 (*completely likely*). The second question asked, “What holds you back from seeking mental health support? (*Check all that apply)” Answers for this item were identified by the GYC and included concerns about confidentiality, stigma, lack of information, lack of parental understanding, nothing, and “not listed.” One open-ended question asked, “What would make it easier for you to access mental health support or resources”? Participants were asked three demographic questions: grade, ethnic/racial identity, and gender identity.

### 2.4. Procedure

This YPAR project used a nonexperimental, transformational survey design. The online, anonymous, self-report survey included quantitative and qualitative (open-ended) survey questions. The GYC collaboratively reviewed the survey questions used in May 2020 (see Rocha et al. [7]) to determine which questions were relevant to their current—2022—research questions. The 2020 survey was shared via email link during remote education due to the COVID-19 pandemic, but the 2022 GYC wanted to change the recruitment strategy because most students were back at school. The local high school began offering optional in-person attendance in April 2021, and by spring 2022, the large majority of students (90–95%) were participating in in-person classes [40]. The GYC met with University partners to discuss research goals and their desire to recruit students to take the survey via a QR code during lunch. Their shift in the research question and recruitment also meant they needed to revise the survey to be much shorter to increase students’ willingness to complete it. We integrated the updates into a revised proposal to the University Institutional Review Board (IRB). As with our prior YPAR study, the IRB determined the 2022 study did not meet the criteria for human studies research due to the anonymity and specificity of the data for the local community. However, we followed ethical procedures per the IRB standards.

The 2022 GYC distributed the online survey via QR code during lunch at their high school between 30 May and 3 June 2022. Youth participants completed informed consent on the first page of the online survey. Parental consent was not sought due to the anonymity of the survey. The school principal approved the survey. Informed consent specified anonymity, voluntary participation, estimated time (<5 min), and names of the collaborators. We shared the intention of the survey was to “(a) identify ways to improve access to mental health resources, and (b) inform adult allies and leaders from the school district and city on how you all are doing”. The last page of the survey included local and national mental health resources. 

The GYC members were leaders in developing the research question, selecting the research design, and developing the survey. City and adult allies who supported this project included: (a) the Gonzales Community Engagement Director, who supports and oversees the GYC; (b) the GYC advisor, who provides professional development and leadership mentoring to the GYC members; (c) the peer mentor, who is a former GYC youth commissioner; and (d) the school principal.

### 2.5. Quantitative Data Analysis

We used IBM^®^ SPSS Statistics (Version 27) to compare 2020 and 2022 responses regarding levels of happiness, depression, and anxiety. We conducted a Mann–Whitney U Test because it allows for the comparison of two sets of data from independent groups with non-normal distributions [41]. Due to significant differences in gender identity between samples and literature indicating adolescent girls are more at risk for depression [42,43], we ran an exploratory analysis (two-way between-groups analysis of variance) to inspect the interaction of year and gender. Lastly, we ran a descriptive analysis in addition to using cut-off scores to review the proportion of youth falling within the PH-Q categories for mild (0–2 on either subscale or a total score of 0–5) and moderate to severe (score of 3 to 6 on either subscale or a total score of 6–12). University partners conducted the initial statistical analysis and brought the aggregated data to the GYC during a data analysis workshop, where we interpreted the data together.

### 2.6. Qualitative Data Analysis

The qualitative data (open-ended response) was separated into a Google Sheet from the quantitative data (e.g., demographics) to allow for collaborative data analysis with the GYC. University partners taught and mentored the thematic qualitative analysis process during a workshop and follow-up meetings [44]. Five GYC members identified themes, clarified definitions, and systematically coded qualitative data. Two undergraduate students (K.E.S. & R.A.-D.) and their mentor (J.L.L.) helped to review, ask questions, clarify, and support the coding process. The researchers and youth reflected on their identities and discussed differing perspectives during the analysis and interpretation process. This reflective process helped reduce bias and value the connection between data and lived experiences. We used pivot tables to identify theme counts. We worked together to draw initial conclusions based on the findings.

## 3. Results

### 3.1. Research Question 1: Comparison of Mental Health 2020 to 2022

Our first research question was, “How does the mental health of Gonzales High School students in 2022 compare to 2020”? Our hypothesis was that the mental health and happiness of Gonzales High School students would be better in 2022 compared to 2020. A Mann–Whitney U Test comparison revealed significantly lower scores of depression and anxiety among students surveyed in 2022 (*Md* = 3, *n* = 229) compared to 2020 (*Md* = 4, *n* = 175), *U* = 15493, *z* = −3.94, *p* < 0.001, *r* = 0.20. Another Mann–Whitney U Test revealed a significant difference in happiness, with students reporting greater happiness in 2022 (*Md* = 7, *n* = 216) compared to 2020 (*Md* = 6, *n* = 174), *U* = 22,371, *z* = 3.257, *p* = 0.001, *r* = 0.17. Our hypothesis was supported, but both effect sizes are small [45]. We also ran a two-way between-groups analysis of variance and found no interaction between gender and year, *F* (1, 377) = 2.73, *p* = 0.10. Significant main effects for gender and year emerged from this analysis, but also with small effect sizes (partial eta squared = 0.02 and 0.03, respectively).

On the descriptive level—using standard cutoff scores for the PHQ-4—approximately half (52%) of the 2020 participants screened at risk for depression and/or anxiety based on their subscale scores, and in 2022 this dropped to 36%. Looking at the overall/total score for mental health risk, 38% of high schoolers scored within the moderate to severe range in 2020, and 23% scored within the moderate to severe range in 2022.

### 3.2. Research Question 2: Access and Utilization of Mental Health Resources

For our second research question, we asked: “How can we improve access and utilization of mental health resources?” To understand areas for improvement, our 2022 survey included two quantitative and one qualitative open-ended question.

**Figure 1:**
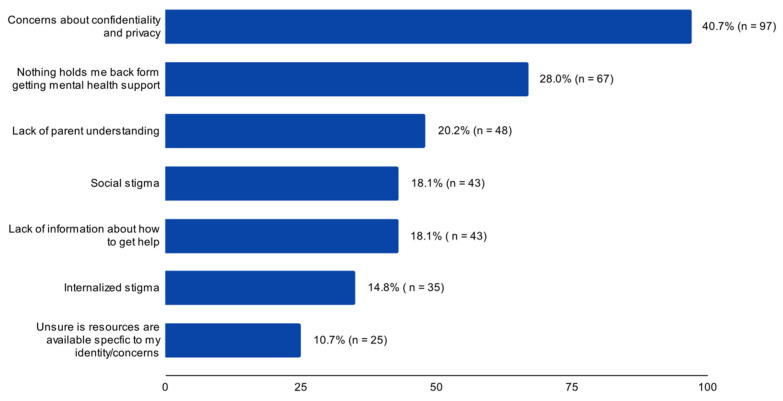
Youth Responses: What holds you back from seeking mental health support? (*n* = 240).

Our first quantitative question asked how likely participants were to seek mental health support; 34.6% (*n* = 83) responded *not at all likely*, 31.7% (*n* = 76) responded *slightly likely*, 22.6% responded *moderately likely*, 7.8% (*n* = 18) responded *very likely*, and 2.1% (*n*= 5) responded *completely likely.* A follow-up quantitative question asked what held them back from seeking mental health support, and participants could select all that applied. The response with the highest frequency was “concerns about confidentiality” (40.7%, n = 97). Following this response, “nothing holds me back”, “lack of parental understanding”, “social stigma”, and “lack of information” were the highest frequency responses from participants. See Figure 1 for details.

In addition to the scaled quantitative results, we also used a qualitative open-ended question to ask what would make it easier for students to access mental health resources and support, and we analyzed this data using thematic qualitative analysis. Through the collaborative qualitative analysis, youth researchers identified 11 thematic codes/themes that we then consolidated into 8 final themes. Of the codable responses, the most prominent themes included: *I don’t know* (24.68%, *n* = 38), *promote awareness and establish resources* (19.48%, *n* = 30), and *normalize and create safe spaces* (18.83%, *n* = 29). First, we found that students expressed a lack of awareness of mental health and did not know what might help to improve access. Students also expressed wanting existing mental health resources to be promoted and better knowledge of where they can go for mental health support on their campus and within their community. Moreover, many students desired the normalization and decreased stigma of mental health challenges and safe environments to be established to comfortably talk about it and access support. See Table 3 for details.

### 3.3. Reflections on the YPAR Process and Youth/University Perspectives

Part of what is transformative about YPAR is the impact it has on researchers and community partners. To honor the reflective process, the authors of this manuscript spent time discussing the impacts of this project on themselves as individuals and as a group. Anyon et al. [31] highlighted the importance of youth outcomes in YPAR work and described youth outcomes for different YPAR projects including social, agency/leadership, emotional, interpersonal, cognitive, academic/career, and critical consciousness impacts. Although not formally measured in this study, we have included youth and University researcher reflections below, organized into common themes from our discussions.

#### 3.3.1. Reflections from Youth

- *Youth Experience Matters*: First, we (A.V.C. & V.C.P.) found through our involvement in GYC and the YPAR process that youth voices and experiences matter. Youth were encouraged to reflect on what mattered to us, we harnessed these insights as a guiding force to navigate the collective challenges we faced. We also recognized that witnessing mental health issues in our lives and the lives of our families compelled us to take proactive steps and initiative on this project. There is a need for youth advocacy for mental health and discussion within the community.
- *Knowledge to Action:* A powerful takeaway from the YPAR experience was that knowledge and data are important for motivating advocacy/action, increasing mental health awareness in our community/school, and identifying gaps and ways to address these gaps. One of our GYC peers expressed gratitude for parental understanding of mental health, but this discussion also illuminated a critical gap—the absence of mental health support for students without family understanding. We learned how “data” includes our experiences and observations in addition to quantitative and qualitative findings from our survey. Multiple forms of data are important to help us recognize challenges with support, and youth-led collaboration is crucial to building up social support for ourselves and future generations.
- *Learning Deeper:* As youth authors, we have been engaged in a journey to explore the mental well-being of our peers, and this journey has been a process of both self-discovery and a deeper understanding of the experiences of Mexican-American youth. Further, understanding the collective mindset, a number of youth and GYC alumni talked about how mental health is often ignored in Latinx/Mexican-American culture. Thus, broadening community understanding of how to identify and refer someone to a mental health professional will help build youth trust with adult allies. We learned from peers and are now interested in learning more from our parents about their perspectives. Parents are influential in finding better resources for their children, but it takes a community to fully address stigma and improve wellness.
- *Professional Skills:* Our experiences in the YPAR project and GYC leadership have given us opportunities to identify potential career paths. We have developed important professional skills by being involved in action projects, researching mental health, giving feedback on the progression of our community center, beautifying student bathrooms, and advocating for more advanced placement classes. Council ambassadors and commissioners participated in a summer fellowship program where we gained hands-on opportunities and received feedback on how to communicate and network with others. In addition to contributing to collaborations, these opportunities offer insight into a more competitive setting, providing chances to acquire skills that ensure confidence and preparedness for college.
- *Relationships and Connections:* An important highlight from our reflections was the intertwined nature of relationships and mental health advocacy. Through our narratives, we experienced a positive impact of fostering connections, whether familial, within the GYC, with professionals in the field, or with youth in other communities who want to learn about our work. We have used these relationships to try to address the pressing need for reliable mental health support in our school. Participating in academic presentations and co-facilitating workshops has allowed us to learn from others and be appreciated for our own expertise. We better understand the power of dialogue and how it can be used to raise awareness. Our reflections emphasized the vital role relationships play in our advocacy journey. In essence, the interplay between personal narratives and collective experiences illustrates how relationships and connections serve as catalysts for change in the realm of youth mental health advocacy.

#### 3.3.2. Reflections from Undergraduate University Researcher-Authors

- *Shared Learning and Scholar Identity:* Throughout our collaboration with the GYC, we (K.E.S. & R.A.-D.) have experienced the shared learning of the mentorship process. Along with conducting research, one of our goals was to develop and nurture a researcher/scholar identity for youth involved in the project. We have seen high school students in Gonzales use this research to advocate for their peers and communicate to community stakeholders (e.g., the superintendent) about their needs. One of the huge benefits of this type of collaboration is the ability to bring shared expertise to the table throughout the research process. Thus, we had to also reflect on our own areas of expertise and areas for growth. Being a mentor involves a shared journey that includes learning from mentees; one example is that we learned from the youth and their advisors ways to improve engagement when facilitating a conversation about research and next steps (e.g., “working the walls” using poster paper to draft ideas). The collaborative learning process helped us to have a more effective and efficient workshop when identifying the next steps for our research.
- *Power of Relationships:* YPAR has shown us the power of relationship building and the joys of mutual healing. We have realized this by recognizing the importance of learning from each other, learning through doing and dialogue. We have worked to center youth voices first to support the vision of change the GYC has for their communities. We joined our mentor’s (J.L.L) research lab in Spring 2022, so we benefited from the foundation of relationships between our mentor, the GYC, and community stakeholders. The established partnership with the GYC accelerated the learning opportunities and provided a theoretical framework and praxis for engaging in this research. As new researchers, we benefited from the built and sustained relationships crucial to the YPAR process, and we were excited to be part of the project’s next phase. Through each of our own mentee relationships with our research mentor and our roles in the mentorship of the GYC, we were able to begin to heal our own struggles with imposter syndrome. We are each healing our own long-held ideas of what it means to be a scholar and researcher. Both from low-income families and first-generation college students, we have found joy in being part of amplifying the voices of students, as our voices are amplified by those mentoring us.
- *YPAR Research Skills and Career Pathway:* This project hinges on shared expertise where everyone involved brings their unique lenses and sets of experiences to the collaboration. We learned to value our lived experiences while valuing youth as community experts. Our team brought validated measures and varying research experiences to help youth clarify their research questions, select research design elements, and identify survey items that helped them answer their research questions. We were able to bring our different backgrounds in psychology (R.A.-D) and public health (K.E.S) to this interdisciplinary collaboration. Participating in this project has been a transformative experience for us and has impacted our future direction as we both plan to conduct YPAR and community-engaged research in graduate school. We also both hope to pursue academic/teaching careers that help students learn these important research skills.
- *Letting Go of Personal Research Desires*: The YPAR approach challenges traditional research dynamics and adult-youth power dynamics by making youth the experts in the research process [8]. As university researchers, we felt discomfort (at first) when letting go of our questions and de-prioritizing our desires for this project when they were not aligned with what the GYC sought to investigate. For brevity and accessibility, the GYC wanted to keep their survey shorter in 2022, and we were unable to add some of the follow-up questions that we were interested in exploring. It was initially challenging to shift from an academic mindset of “what do I want to know” to the community-based mindset of “what do youth want/need to know”? Letting go of personal research desires for the sake of the YPAR process took restraint and respect for the YPAR approach.
- *Scheduling and Communication Challenges:* The biggest challenges we encountered in our YPAR experience were largely logistical, including scheduling, communication, and timeline. Everyone involved in this project has busy schedules including family, school, work, and extracurricular commitments, making finding compatible meeting times challenging. We were used to conducting some meetings via Zoom, but the new group of GYC members was not as engaged virtually. As a result, we decided to shift to some in-person meetings/workshops. During the workshop, there seemed to be great momentum, but then there was a lack of follow-up and responsiveness to emails. Thus, we struggled to gauge levels of interest and capacity from the GYC members and wanted to avoid being too directive/urgent with our own optimal timeline. While virtual communication tools benefit from making partnerships possible and sustainable with busy schedules, we found that it was more challenging to build authentic relationships online than in-person meetings. As University partners in this work, we sometimes struggled with the balance of accountability for communication and letting the youth initiate communication.

## 4. Discussion

Quantitative and qualitative data from this YPAR project provides insights into the mental health of Latinx/Mexican-American youth from an agricultural city during and following the COVID-19 pandemic. Our research questions sought to understand the mental health of Gonzales High School students in 2020 compared to 2022, their willingness to seek support, and what would make it easier for youth to access and use mental health resources. Although effect sizes were small, our hypothesis was supported in that the risk for depression and anxiety decreased from May 2020 to May 2022, and reported happiness increased. Despite an increase in mental wellness among the 2022 cohort of high schoolers, approximately one-third of students were still at risk for anxiety and/or depression. However, more than a third of participants reported they were “not at all likely” to seek help if experiencing a mental health challenge. The most frequent barriers and concerns endorsed by youth included: concerns of confidentiality, lack of parental understanding, and stigma.

When asked what could improve access, the youth’s qualitative responses suggested promoting awareness, establishing resources, and normalizing mental health by creating safe spaces. Interestingly, the qualitative data also revealed that 24.68% (*n* = 38) of high school participants did not know what mental health resources were available or what might be needed to improve access. This aligns with past research on Latinx mental health, which has found a lack of knowledge and cultural stigmas often prevent Latinx individuals from accessing mental health support [13]. This was also an important topic discussed by GYC youth during our analysis and interpretation workshops as they were connecting the results to their own lives. These results document some challenges related to participant’s awareness of mental health resources and their likelihood to utilize available resources.

The data comparison between 2020 and 2022 is encouraging, demonstrating a decrease in mental health risk among youth. However, there were small effect sizes and adolescent mental health risk remained high. This local trend of high mental health risk is in line with other research assessing national youth mental health risk described in the Youth Risk Behavior Survey [33]. Although there were some studies indicating initial stress and decreases in wellbeing when students returned to in-person school within the few months following the shutdown [46], the gap between return (spring 2021) and the timing of this follow-up (May 2022) seems to have allowed for some time to see improvements, albeit small. GYC youth discussed the importance of social integration and access to campus support helping improve their mental health.

This research is important because the Latinx/Mexican-American population is the fastest growing population in the U.S., yet confronts challenges in mental health and barriers to accessing supportive resources. Thirty percent of Gonzales’ population is under 18 years of age [47], and 93% of high school participants identified as Latinx/Hispanic/Mexican-American. It is powerful to have Mexican-American youth researching mental health and advocating for what is important to them. Mental health research is often led by people in academia, and this project is unique because it is led by—and shared by—youth with the support of adult allies/partners. Due to the recent nature of the COVID-19 pandemic, research is still relatively new, and there is emerging data available on the long-term effects on mental health [48]. Involving Latinx youth in healthcare research at this time is particularly important.

### 4.1. Action and Advocacy Outcomes

Shifting knowledge into action through the YPAR process has the power to create change by identifying the challenges youth and their communities face. To help promote mental health literacy and awareness, the GYC engaged in action and advocacy efforts based on our 2022 findings. First, two GYC leaders (including A.V.C.) worked with undergraduate researchers to summarize the data and co-present via two academic posters. We also co-presented this research as a symposium at a national conference to promote the use of YPAR and demonstrate the power of research produced by youth for youth mental health.

After diving deeper into the data to create a research poster, the fourth author (A.V.C.) worked with peers to organize an event to share the findings with the community of Gonzales. Our goals were to show our community the work the GYC has done, hear feedback on what we could work forward on, and get the input of parents, community service workers, and/or other students about mental health. We held the event in the library of Gonzales High School (GHS) and titled it *Inside the Minds of GHS*. The goal of this event was to connect with our community about mental health, talk about our data, and seek community ideas they wanted to share or reactions to the data. GYC members shared the research findings and engaged the community in discussions about mental health. Approximately 15 people attended the event, and we were particularly grateful for the fire chief and the local superintendent to be present. The GYC leaders spent a few weeks preparing and planning the event. Fewer people attended than we had hoped, but we created a safe space and a bond to share another experience and ideas about mental health. Overall, there was a feeling of enjoyment and inspiration all in one room, one space, and one meeting. These efforts resonated at Gonzales High, resulting in increased mental health awareness for those who attended and some ideas for how the GYC could help share resources on campus. We connected with the new superintendent at this event and committed to hosting a Mental Health Resources Fair at Gonzales High School in spring 2024.

As a result of our local work, the GYC and University partners were invited to present as part of the inaugural Monterey County Youth Mental Health Summit hosted by NAMI Monterey County, The Epicenter, and The Village Project (7 October 2023). Through meetings and extensive practice, we prepared a workshop to help other youth and community members transform mental health questions into actions. The second (R.A.-D), third (J.L.L.), and fifth authors (V.C.P.) played a pivotal role in the Gonzales Youth Council’s initiative to share research data at this event. The aim extended beyond a conventional presentation, as the GYC sought to provide an engaging and interactive experience for the audience. Incorporating hands-on, collaborative activities, the presentation aimed to inspire participants to critically think about their community’s mental health challenges. Furthermore, attendees were encouraged to establish their commitments to actionable steps, fostering a sense of empowerment and personal investment in the collective journey toward improved mental health.

The GYC’s work on mental health has received local and national recognition. The GYC was featured as a front-page story for the Monterey County Weekly Newspaper [47], and their work has been used to help develop youth councils in other local cities. Gonzales won the 2022 Ruth Vreeland Award for Engaging Youth in City Government (via the Helen Putnam Awards), and was invited as a finalist for the All-America City Award through the National Civic League, a nonprofit promoting civic engagement to address local issues. Two GYC representatives (including the fourth author, A.V.C.) traveled to Denver, CO, to share their research and GYC projects. I (A.V.C.) knew this experience would be thrilling but also frightening as I would be presenting the Mental Health Project data and accomplishments of the GYC. I felt I had to make sure I got everything right. The whole experience was an honor, and it was amazing when we learned that Gonzales was one of 10 cities nationwide to receive the award. Our mental health and other projects inspired people who have not even been to Gonzales and do not know our community. The GYC’s journey, from its inception to the present, has left a lasting mark on the landscape of mental health initiatives within our community and beyond.

### 4.2. Implications for Healthcare Policy and Practice

YPAR can help identify healthcare barriers and inequalities affecting young people and contribute to developing systems that support health equity. Healthcare policy and practice can be more responsive to the specific needs of historically marginalized and underserved youth populations by involving youth in the research and decision-making process. Youth researchers can also identify effective strategies for reaching their peers with health information to promote health literacy, prevention, and promotion strategies. 

In past studies, YPAR has been used in community health settings to address a wide range of health issues, such as access to physical activity options and healthy food, concentrated tobacco and liquor store density in low-income neighborhoods of color, and exposure to hormone-disrupting chemicals in pesticides [8]. YPAR has also been implemented in regional suicide prevention efforts [49]. To utilize YPAR as a health intervention strategy, Lindquist-Grantz and Abraczinskas [49] identified best practices for design, implementation, and evaluation. Some of these practices include early and open communication between academic and community stakeholders and alignment of YPAR practices with the health behavior model. For implementation, the authors recommend facilitators remember to prioritize benefits for youth and the community and balance their own work benefits. The research and change efforts should be aligned with a specific health topic, and the evaluation process should utilize both qualitative and quantitative methods and gain diverse perspectives. Outcomes should focus on youth development, individual health, and long-term community-level outcomes. YPAR can reach a broader population of young people in community-based research for health promotion, and there are free online resources for learning more about YPAR processes [50,51]. 

### 4.3. Limitations and Challenges

Some limitations of this project include using a convenience sample of youth gathered during high school lunch, with a 29.7% response rate (out of 838 enrolled students) [40]. Youths who were not present or missing lunch did not have access to the survey, and some youth declined to be involved. Also, a small proportion of students were still engaged in remote education and were not included. The 2020 and 2022 samples included different students and had different recruitment. The results have limited generalizability due to the sample being 93% Latinx/Mexican-American and from a small agricultural community. However, we consider this a strength rather than a limitation because the purpose of the YPAR project was to gather locally relevant data, and this population is also understudied. Regarding measurement, a limitation of our study is the use of the PHQ-4 as a measure of depression and anxiety due to this measure not being developed for adolescents. However, there are studies supporting the reliability and validity of this measure among adolescents [35,36], and the measure had high internal consistency in our 2020 and 2022 samples. Also, our project provides an example of how validated screeners—often used in healthcare settings—can be used within YPAR research when they align with research questions and are chosen by youth. 

Part of our research team’s ongoing conversation about this project included challenges around ethics and institutional processes. This study was deemed non-human subjects research due to anonymous data, with results specific to the local community. The initial purpose of the study was to gather information during the public health crisis of COVID-19, which IRBs often considered as non-human subjects as well. Grappling with our research as “non-human subjects research” while working with humans and conducting research felt like a paradox. We continually discussed how to ground our work in research and social ethics. At the beginning of the project in 2020, the GYC and our University partner (J.L.L) worked on a memorandum of understanding to establish GYC data ownership. Thus, based on our work, we have sought youth approval, leadership, and involvement in our presentations and publications. The GYC also sought review and collaboration with other adult allies in the school and city. To conduct this survey with high ethical standards, the GYC survey included youth informed consent, non-triggering and low-risk questions, the choice not to answer or opt-out at any point, and a debrief page with mental health resources.

## 5. Conclusions

YPAR is a valuable tool for empowering youth voices, listening to their needs, and advocating for community change. When considered together, our reflections from youth and interdisciplinary University team members highlighted the impact of relationships and the joys of building connections throughout this project. Involvement in this ongoing YPAR project has informed many individual paths into research and further education in undergraduate and graduate programs. Our next steps include advocacy for decreasing mental health stigma in the local community, improving mental health literacy, finding creative ways to increase knowledge of high school resources in Gonzales, and disseminating research findings to community stakeholders. We are also in the process of planning a third phase of our iterative mental health research project. On a broader scale, we hope our work inspires others in healthcare to consider using YPAR and to develop opportunities for youth to be co-researchers and leaders in youth mental health research.

In summary, our YPAR project and process provide an example of how this method can amplify youth voices and inform positive change for youth mental health. Using YPAR can complement large-scale public health research by providing local perspectives and mobilizing youth to take immediate community action. 

## Figures and Tables

**Table 1:** Overview of Youth Participatory Action Research (YPAR) Principles.

YPAR Principle	Ways to Enact Principle
Collaboration	Build authentic youth-adult relationships Share power and decision making Collaborate with youth throughout the research process (e.g., research questions, research design, data collection, analysis, dissemination) Partner with the community and school stakeholders
Empowerment	Value youth expertise and knowledge about inequities Investigate and take action around issues that matter to youth researchers Leverage youth and adult researcher strengths and knowledge Amplify youth voices and needs
Subjectivity	Value-situated knowledge and openness Reflect on the ways researcher identities impact the partnership and research study
Deconstruction	Deconstruct and challenge systems of oppression impacting youth (e.g., racism, sexism, adultism, ableism) Critically examine the perspectives and needs of diverse and marginalized youth Conduct research that disrupts the status quo Have conversations about power and privilege
Transformation	Transform policy and practice to improve the lives of participants and communities Use research for action, advocacy, and allyship Make lasting community changes that support greater equity and social justice in schools and communities Co-create solutions informed by research and feedback loops for continued improvement and dialogue

Note. Table reprinted from Rocha et al. (2022), “Using youth-led participatory action research to advance the mental health needs of Latinx youth during COVID-19”, *School Psychology Review* [7]. Reprinted by permission of the publisher (Taylor & Francis Ltd., http://www.tandfonline.com, accessed on 7 February 2024).

**Table 2:** Sociodemographic Characteristics and Chi-Square Comparisons Between 2020 and 2022 Samples.

Characteristic	2020 (*n =* 176)	2022 (*n =* 234)	*χ^2^*	Effect Size
School Grade			52.8 **	Cramer’s V = 0.36, medium
9th	30 (17.0%)	62 (26.5%)	*	
10th	67 (38.1%)	29 (12.4%)	*	
11th	36 (20.5%)	106 (45.3%)	*	
12th	38 (21.6%)	35 (15.0%)		
Other	5 (2.8%)	2 (0.9%)	–	
Gender			10.2 **	Phi coefficient = −0.17, small
Female	112 (63.6%)	104 (44.4%)	*	
Male	60 (34.1%)	111 (47.4%)	*	
Not listed/ Non-conforming	4 (2.3%)	19 (8.1%)	–	
Ethnicity/Race			0.62	
Latinx/ Mex-Am/Hisp.	162 (92.0%)	218 (93.2%)		
European/White	6 (3.4%)	5 (2.1%)		
Multiracial/ Multiethnic	5 (2.8%)	3 (1.3%)	–	
Other	3 (1.7%)	8 (3.4%)	–	

Note. ** p ≤ 0.001. We set the *p*-value = 0.01 for statistical significance to account for multiple comparisons. For Pearson’s Chi-Square, the * asterisk designates which groups were significantly different based on an adjusted residual ≥ 2.0 or ≤−2.0.

**Table 3:** Youth Responses: What would make it easier for you to access mental health support or resources? *(N* = 154).

Emergent Themes	Definition and Example Quote	Frequency, n(%)
I do not know	Not informed about mental health resources or unaware of what might improve access.	38 (24.68%)
Promote awareness and establish resources	Desire for on campus resources (mental health electives, mindfulness) and promotion of resources to students. “To make it easier to access mental health support or resources I would say to make them more available or to let others know the support is there”.	30 (19.48%)
Normalize and create safe spaces	Normalize mental health challenges by having open conversations and learning about mental health. Decrease stigma for seeking help by creating safe spaces where students are comfortable sharing. “Make it a safe environment in which it makes it safe to seek help without being told we are overreacting or do not have any reasons to feel like we should”.	29 (18.83%)
Nothing	Respondents felt they knew how to access support. They stated nothing would make it easier, and/or they do not need it. “Nothing to be honest and I do not really know cause I am not in a mental health issue right now”	27 (17.53%)
Electronic and quick resources available	Improving access by including more online and website resources or consistent check-ins. “having to make a website and sign up to get called out of class and have a talk with someone”	20 (12.99%)
Confidentiality	Having someone to talk to who will keep information private. “It would be easier for me if I was anonymous and my information would not be shared with anyone, especially my parents”.	17 (11.04%)
Education on mental health for community	Peer support, parents, coordination of services with family when desired by youth. “educating more parents to understand what mental health is and the benefits from taking part in it”.	9 (5.84%)
Community resources	Community-based resources and therapists in Gonzales; mental health resources offered separate from school. “Try bringing mental health specialists that are not involved nor work with the school whatsoever. Only using this as an option to help keep students more comfortable”.	8 (5.19%)

Note. Some students wrote responses or words that were not relevant to the question and/or were unable to be coded (*n* = 49); thus, 203 participants wrote a response, but the number of codable responses was 154.

## Data Availability

Data for this project is owned by the youth council and not shared due to a desire to keep data private. Also, participants did not consent to their raw data being shared beyond the intentions outlined for this project.

## Supplementary Materials

The following are available online at https://www.mdpi.com/article/10.3390/healthcare12050592/s1, Author Biographical and Reflective Statements.
