# Supplementary material for: Youth Are the Experts! Youth Participatory Action Research to Address the Adolescent Mental Health Crisis
Source: Healthcare (Basel). 2024 Mar 5;12(5):592. doi: 10.3390/healthcare12050592 (PMC10930985; doi:10.3390/healthcare12050592)
Supplement: Supplementary file 1 [file healthcare-12-00592-s001.zip › healthcare-2776004-supplementary.pdf]

Smith, K.E.; Acevedo-Duran, R.; Lovell, J.L.; Castillo, A.V.; Cardenas Pacheco, V. Youth Are the Experts! Youth Participatory Action Research to Address the Adolescent Mental Health Crisis. *Healthcare* 2024.

Supplemental Material
Author Biographical and Reflective Statements

Kimberly E. Smith, is an undergraduate Collaborative Health and Human Services major with a Biology minor at California State University Monterey Bay, graduating in May 2024. She identifies as a white, cisgender woman and is a proud first-generation college student from a low-income family in a small rural southern California town. She has been a member of Dr. Lovell's Child Health and Wellness Lab since January 2022. Her research interests involve maternal and child health, social determinants of health, and protective factors for health outcomes. She aims to use community-engaged research to evaluate health disparities and promote positive maternal and child health outcomes for low socioeconomic status populations. She is a McNair Scholar and Sally Casanova Scholar and will begin a Master of Public Health program in the fall of 2024.

Rosa E. Acevedo-Duran, is an undergraduate Psychology major with a minor in Social Justice and Community Leadership at California State University, Monterey Bay. She is a McNair and Sally Casanova scholar, and her primary research interests are youth mental health and wellbeing, critical consciousness, and liberatory healing practices. She identifies as a first-generation Mexican-American from a low-income background in the Salinas Valley and is the oldest of six. She aims to use her research background in community-engaged research to address the disparities faced by underserved people in her community. She has been accepted and will start a Ph.D. in Counseling Psychology in the fall of 2024.

Jennifer L. Lovell, PhD, is an Associate Professor of Clinical Psychology at California State University Monterey Bay (CSUMB). She completed her Ph.D. in Clinical Child Psychology at Southern Illinois University, Carbondale, and later specialized in pediatric psychology and infant mental health during postdoctoral training at the University of Colorado School of Medicine. Her primary areas of scholarly interest focus on social and cultural factors impacting youth health and wellness, and she highly values her role as research mentor to undergraduate students and young people. She is author of a book focused on using a strengths-based approach to clinical work with adolescents from multicultural backgrounds (*The "Troubled" Adolescent: Challenges and Resilience within Family and Multicultural Contexts*; Lovell & White, 2019). Dr. Lovell identifies as a White, multi-ethnic, cisgender woman from a middle-class family who has lived in the Monterey Bay area since 2016. She aims to use her research to address local community needs and improve young people's lives.

Aliyah V. Castillo, is a Senior at Gonzales High and has been a member of the Gonzalez Youth Council since 2020. She currently serves as one of two Youth Commissioners for the 2023-2024 term. As a first-generation Mexican-American, she actively participated in various action projects, including the beautification project, pedestrian safety, and gained prominence in the Mental Health Project. Each project provided her with opportunities for personal growth and the development of valuable skills. The Mental Health Project, in particular, inspired her to advocate for her peers and community members. The knowledge Aliyah has acquired and applied has motivated her to continue her journey. She plans to pursue further growth and knowledge by majoring in psychology, with the intention of attending either a community college or a four-year university. Her ultimate goal is to become a child psychologist or social psychologist, contributing to the well-being of children and communities.

Valeria Cardenas Pacheco, is a Junior at Gonzales High School and has been part of the Gonzales Youth Council since 2022. Valeria is currently serving as a youth Ambassador for the 2023-2024 year. She identifies as a first-generation, low-income Mexican-American born to migrant farmworker parents, raised in Gonzales, CA. As one of the three youth ambassadors for the Gonzales Youth Council, she helps to lead a youth run council of 15 members advocating for their community needs in both our city and school. Beyond her commitment to social progress, Valeria finds solace and inspiration in the world of literature and firmly believes in upholding honesty and moral principles. In pursuing her dream of attending a four-year institution and majoring in international relations, she aspires to become a policy attorney, dedicated to creating positive change on a global scale.
